# Comparison of perceptions of domestic elder abuse among healthcare workers based on the Knowledge-Attitude-Behavior (KAB) model

**DOI:** 10.1371/journal.pone.0206640

**Published:** 2018-11-01

**Authors:** Qinqiuzi Yi, Naohiro Hohashi

**Affiliations:** Health Sciences, Kobe University, Japan; Nord University, NORWAY

## Abstract

It is generally agreed that healthcare workers are ideally positioned to recognize and diagnose cases of elder abuse. However, little is known about their knowledge and understanding of this issue. The objective of this study was to assess and compare the perceptions of different groups of healthcare workers toward elder abuse in Japan, using the Knowledge-Attitude-Behavior (KAB) model. Home-visit nurses, medical doctors, care managers, care workers, public health nurses, and social workers, with experience of dealing with elder abuse received self-administered questionnaire surveys that inquired regarding demographics, knowledge, attitudes, and behaviors regarding elder abuse. A total of 311 healthcare workers participated in this survey. To compare the differences among the groups, a one-way analysis of variance with a post-hoc Tukey’s test, and a Kruskal-Wallis with post-hoc Steel-Dwass tests were used in accordance with data normality. Multiple linear regression analysis was conducted to explore variables that predicted the healthcare workers’ perceptions, and covariance structure analysis was used to examine whether the KAB model can accurately predict healthcare workers’ perceptions. Multiple comparisons showed significant differences in knowledge, attitudes, and behaviors regarding elder abuse among the abovementioned six groups. Age, sex, and years of work related to the care of elderly were extracted as significant determinants of healthcare workers’ perceptions of elder abuse. The examination of the KAB model with covariance structure analysis yielded a model with strong goodness-of-fit. These findings emphasize the need to take effective measures to improve their perceptions as well as review the role of each healthcare worker so that they can be more concerned with and involved in the safeguarding of the elderly. Given the strong goodness-of-fit demonstrated by the KAB model, education of healthcare workers on both the knowledge of, and attitudes toward, elder abuse may help in improving healthcare workers’ behavior in dealing with elder abuse.

## Introduction

As population aging advances, a variety of issues and challenges have arisen concerning care of the elderly, with elder abuse attracting considerable attention. Recent estimates of the prevalence of elder abuse in Japan, including those residing in care facilities and those living at home, range from 15.4 to 34.9% [1–4], depending on the research methodology of the questionnaire survey or with different study samples such as elderly themselves or family caregivers. Unfortunately, prevalence reports may underestimate actual incidence because many cases of elder abuse are still unidentified and unreported [5].

Similar to the World Health Organization’s stance on elder abuse, Japan’s Elder Abuse Prevention Law (full name: “Act on the Prevention of Elder Abuse, Support for Caregivers of the Elderly and Other Related Matters”) categorizes physical abuse, psychological abuse, sexual abuse, economic abuse, and neglect as subtypes of elder abuse. However, as Takeda pointed out, beyond these subtypes, other problems such as human rights violations and inappropriate care of the elderly may also occur in Japan [6], such as elder self-neglect, which means the falling of elderly people into a state that will endanger their safety and physical condition, due to either refusal or inability to perform actions that would normally be carried out in life by the elderly person himself/herself [7]. In addition there is social abuse, in which the elderly person’s social contact with others was cut off, or restricting the elderly person’s social activities to socially isolate him/her, making him/her feel socially excluded [8]. Therefore, to obtain a comprehensive understanding of this issue, it is necessary to include elder self-neglect and social abuse as forms of elder abuse.

Elder abuse nearly always occurs in non-public venues such as in private homes. In Asia, because of cultural influences, abused elderly is often reticent about disclosing cases of abuse to avoid besmirching the honor and dignity of their family [9]. Consequently, elder abuse, especially when occurring in private households, remains a serious challenge in Asian countries. As the study pointed out, in Japan, elderly who have physical and mental illness/disability, whose ability of judgment and self-care have declined, are at a higher risk of domestic elder abuse, and the reasons for domestic elder abuse in Japan mainly lie in problematic human relationships between caregiver and the elderly person [10].

Healthcare workers such as home-visit nurses, medical doctors, and care workers play an important role in identifying, reporting, and preventing elder abuse because their work takes them into the elderly person’s living environment. Here, they can grasp the existence and circumstances of elder abuse, and most elderly individuals trust them. However, as it has been acknowledged that the identifying, documenting, and reporting of cases of elderly abuse are not carried out consistently among healthcare workers [11], this insight reflects a current situation that, although healthcare workers are on the frontline in cases of elder abuse, they may not be actively grappling with this issue with appropriate levels of awareness or training.

During the last 15 years, studies have shown that medical doctors believe elder abuse is not a problem in their patient populations [12], or that reporting it may influence rapport with their patients [13]. Health and welfare practitioners working with elderly who experience abuse face such challenges as intimidating context, practice dilemmas, and lack of support—thus perceiving the work itself as difficult, complex and at times dangerous [14]. From the above, we suggest that one of the reasons why the healthcare workers did not actively provide for identification or prevention of elder abuse might lie in healthcare workers’ perceptions thereof. Moreover, nowadays in Japan, health and welfare specialists have established networks, such as the team specializing in dealing with domestic elder abuse through the cooperation of the Japanese Association of Certified Social Workers (JACSW) and the Japan Federation of Bar Associations (JFBA), to reduce abuse of elderly. However, Nakanishi et al. pointed out that the development of intervention teams and the establishment of networks have not achieved much progress [15], and further policy should still address how to establish intervention teams and multi-agency networks. In order to improve the establishment and development of multi-disciplinary collaboration teams for coping with elder abuse, the first step, we suggest, would be to determine to what extent different types of healthcare workers recognize this issue, as well as comparing their perceptions of elder abuse. This may assist healthcare workers to understand their perceptions toward elder abuse, and also help them review their role in dealing with elder abuse.

The Knowledge-Attitude-Behavior (KAB) model, which is developed as a health promotion model and frequently used to assess behavior change, has been proposed as a way of explaining the role of knowledge. It explains that a person’s knowledge directly affects his/her attitudes, and indirectly affects behaviors through his/her attitudes [16]. With this model, it can be assumed that the knowledge of healthcare workers about elder abuse affects their attitudes toward it, and that their attitudes affect the actions (behaviors) they take. In addition, the Family Care/Caring Theory (FCCT), proposed by Hohashi, underlines the importance of behavior and attitudes when healthcare workers are conducting family interventions. It explains that not only is it important to take action to maintain and improve the family’s well-being, but it is also critical to have the attitude to understand family beliefs, intentions, and hopes and to use these in family care [17]. Building on these insights, we argue that it is necessary to assess the perceptions of elder abuse by healthcare workers from the aspects of knowledge, attitudes, and behaviors. Altmann pointed out that attitude is bipolar, and can be positive or negative, favorable or unfavorable [18], so we therefore consider that the attitude toward knowledge of elder abuse can also be positive or negative. A positive attitude toward knowledge of elder abuse implies better knowledge toward the problem of elder abuse, willingness to deal with it, and a state of mind that envisions and expects favorable results from the response to elder abuse. On the other hand, a negative attitude toward knowledge of elder abuse implies unfamiliarity with the problem of elder abuse, unwillingness to deal with the problem, and a mindset that the results of responding to elder abuse will be unfavorable. For this study, we decided to use the KAB model as a research model; however, the KAB model is usually used to assess behavior change, and whether the relationships within the model can really predict healthcare workers’ perceptions of elder abuse is still unknown; verification of the KAB model as applied in this way may still be necessary.

Above all, the main objectives of this study were to: 1) assess and compare the perceptions of healthcare workers toward domestic elder abuse in Japan in terms of knowledge, attitudes, and behaviors; 2) explore demographic variables that predict the healthcare workers’ knowledge, attitudes, and behaviors; and 3) examine whether the KAB model can predict healthcare workers’ perceptions of elder abuse.

## Methods

This study was reviewed and approved by the institutional review board of the researchers’ university (approval number: 587).

### Study samples

We decided to choose home-visit nurses, medical doctors, care managers, care workers, public health nurses, and social workers as study sample groups. These were based on the points of view that, firstly, the home-visit nurse has the role of providing nursing and care services, and supports recuperation in people afflicted with disease or disabilities who live at home. On the other hand, care worker, whose job position requires national qualification in Japan, mainly engages in physical care and provides living support for people afflicted with disease or disabilities who live at home. These roles suggest that the home-visit nurse and care worker can immediately assess the presence of elder abuse and its circumstances. Secondly, according to the most recent nationwide survey (2016) conducted by Japan’s Ministry of Health, Labour and Welfare, the largest number of whistleblowers of cases of elder abuse were care managers, who in Japan hold such qualifications as medical doctor, or nurse, and who have at least five years of practical experience. Care managers, who accounted for 29.5% [19] of the total, were the largest in number, an indication that care managers play a significant role in the discovery of cases of elder abuse. They also perform the role of consulting with a person who needs care, as well as creating care plan for them. Thirdly, public health nurse is a national qualification in Japan whose role is prevention of diseases and promotion of public health through local activities and health education. A social worker is a specialized profession entasked with building relationships with people undergoing difficulties in life; people suffering from anxieties in life; and people who have been socially alienated; and provides assistance to resolve those problems. In particular, public health nurses and social workers who work at Comprehensive Community Care Centers (which have functions including ranging from the delivery of preventive care to the management of difficult cases, and aim to provide generic support for improvement of medical health and welfare) have important roles in supporting health and life of the elderly in the community, and function in cooperation with various local health, medical, and welfare organizations. Lastly, medical doctors have the role of conducting physical assessments of the elderly, and also most elderly tend to trust them, placing them in a better position of discerning signs of elder abuse.

To assess and compare the perceptions of six groups of healthcare workers toward elder abuse, power analysis with G*Power (version 3.1.9.3) revealed a desired sample size of 216 participants, with each group consisting of 36 participants. Research participation requests and research questionnaires were delivered to home-visit nursing stations that also included the home care support centers in order to recruit home-visit nurses and care managers (five questionnaires per station). These mailings were also sent to the home care facilities to recruit care workers (one questionnaire per facility), to the supporting clinic of home health care facilities to recruit medical doctors (one questionnaire per facility), and to the Comprehensive Community Care Centers to recruit public health nurses and social workers (two questionnaires per center). Study samples that met the inclusion criteria of having experience related to the care of the elderly, and who did not meet the exclusion criteria of no experience in dealing with elder abuse, were asked to complete a self-administered questionnaire. The data were collected between November 2017 and February 2018.

### Operational definition of basic terms

The definition of knowledge, attitude, and behavior used in this study was as stated below.

1. **Knowledge**: Knowledge can be generally defined as a justified true belief [20], and for the purpose of this study, we defined it as a belief that the perceived elder abuse is true and accurate. The beliefs are obtained through experience, education, etc., and form the foundation for judgment of elder abuse.
2. **Attitude**: Attitude refers to a response to a stimulus. It is bipolar, and has cognitive, affective, and behavioral components. The cognitive component is an evaluation of the entity that constitutes an individual’s opinion (belief/disbelief) about the object. The affective component is the emotional response (liking/disliking) towards an attitude object. Finally, the behavioral component refers to a verbal or overt (nonverbal) behavioral tendency by an individual, and consists of actions or observable responses that are the result of an attitude object [18, 21]. In this study, we defined attitude as a response to elder abuse, has positive and negative sides, and has cognitive, affective, and behavioral components.
3. **Behavior**: According to Hohashi’s definition, behavior is the way a person subconsciously or consciously acts or behaves towards someone or something [22]. In this study, we used it as the way a person acts and behaves with the aim of eliminating abuse of the elderly.

### Measures

Participants completed a self-administered questionnaire on demographic details, and questions related to elder abuse. Completing the questionnaire required approximately 15 minutes. Demographic items included age, sex, occupation, workplace, years of work related to the care of the elderly, total years of experience, education level, and whether they have attended courses on elder abuse. Questions related to elder abuse were composed of: (1) knowledge about elder abuse and its acts; (2) attitudes toward elder abuse; and (3) behaviors in relation to dealing with elder abuse. Because this study was largely exploratory in nature, we created our own questions and response options based on our previous studies [10, 23, 24]. All questions were designed based on professionals’ clinical experience coupled with broader information regarding elder abuse from existing literature. Healthcare workers selected from the home-visit nursing stations (numbering 240 for questions related to knowledge and 39 for questions related to attitudes and behaviors) evaluated the face and content validity of the designed questions, and final changes to the questions were made based on their input. The contents of the questions are outlined below. The Cronbach’s α of the knowledge questionnaire was .88, broken down into .61 for the attitudes questionnaire and .96 for the behaviors questionnaire, thus confirming the reliability of these questionnaires.

1. **Knowledge about elder abuse and its acts** [23]: Thirty-four questions, with response options of “*physical abuse*,” “*neglect*,” “*psychological abuse*,” “*sexual abuse*,” “*economic abuse*,” “*social abuse*,” “*self-neglect*,” “*do not think it is elder abuse*,” and “*unknown*,” were used to ask the respondents to assess whether the item was appropriate as an act of abuse, as well as choose the item’s subtype of abuse. Higher scores indicate more abundant knowledge concerning elder abuse.
2. **Attitudes toward elder abuse** [24]: Twenty-eight questions were used to ask the respondents to evaluate to what extent they thought about attitudes toward elder abuse, based on a 5-point Likert scale from “*strongly agree*” to “*strongly disagree*.” Higher scores indicate more positive attitudes toward knowledge of elder abuse.
3. **Behaviors regarding dealing with elder abuse** [10]: Thirty-nine questions, with response options on 4-point Likert scale from “*always do*” to “*do not do*” and an option of “*think that it is necessary*, *but outside the scope of my role*,” were used to ask the respondents to choose what behaviors they have adopted regarding dealing with elder abuse as well as assessing its frequency. Higher scores present adoption of behaviors toward dealing with elder abuse in a more positive manner.

### Data analyses

SPSS software (version 18.0) and R software (version 3.4.3) were used for statistical analysis. The demographic data of the study participants were summarized using descriptive statistics and expressed as mean (standard deviation, SD, in parentheses) or total number (percentage in parentheses). Data normality was initially verified using the Kolmogorov-Smirnov test. Differences in the demographic data among groups were analyzed using the chi-squared test for categorical variables, and one-way analysis of variance (ANOVA) or the Kruskal-Wallis test (non-parametric equivalent of the ANOVA) for continuous variables, in accordance with the data normality. To analyze differences of knowledge, attitudes, and behaviors among the different occupational categories, Tukey’s tests were used for post-hoc analysis of the parametric variables analyzed using the ANOVA, and post-hoc comparisons for the non-parametric variables analyzed using the Kruskal-Wallis test were made Steel-Dwass multiple comparison tests. Multiple linear regression analysis with the stepwise method was adopted to explore the influence of demographic factors on the study participants’ knowledge, attitudes, and behaviors about elder abuse. Dummy variables were established for sex (with “male” as reference), for attended courses on elder abuse (with “no” as reference), and for occupational categories (with “medical doctors” as reference). In addition, to examine the KAB model, covariance structure analysis was conducted and the model was identified by ascertaining the path directions, standardized estimates, goodness-of-fit index (GFI), adjusted GFI (AGFI), comparative fit index (CFI), Bentler-Bonett normed fit index (NFI), and root mean square error of approximation (RMSEA). The model was judged to fit the data well if GFI, AGFI, CFI, and NFI were greater than or equal to .90 and RMSEA was less than or equal to .08. The required significance level (alpha) for all tests was .05.

### Ethical considerations

This study was structured as an anonymous, self-administered questionnaire survey. All study participants were received a research participation request that explained the objectives and significance of the study. They were also informed that participation was voluntary and that names and personal details would not be included in the study, and no incentives were offered for participation. Agreement to participate in the survey was indicated by checking the relevant box in the questionnaire. Returning the completed questionnaire to the researchers constituted agreement to participate.

## Results

A total of 374 questionnaires were successfully collected, and the approximate response rate was 33.2%. The accurate response rate could not be precisely determined because the specific number of questionnaires distributed to the healthcare workers at the sites was unclear. Assuming that all the questionnaires had been distributed to the healthcare workers, the response rate may be higher than 33.2%. Responders were excluded where they did not provide demographic characteristics (13 individuals); did not respond to at least 10% of all questions (seven individuals); or whose professions—physical therapists or occupational therapists, for example—were not included in the six types in the study sample (43 individuals). Valid responses were obtained from 311 study participants, consisting of 60 home-visit nurses, 44 medical doctors, 51 care managers, 47 care workers, 46 public health nurses, and 63 social workers.

### Sample characteristics

Sample characteristics are presented in Table 1. Among all the participants, the percentage of males was 31.9% and females 68.1%. Significant differences were found in the ratio of males to females in the six groups. Participants working as medical doctors were the oldest, had the most years of work related to the care of the elderly, and the highest total years of experience. The educational background for the majority of the participants was that of a four-year/six-year university graduate (43.1%), and this was followed by those who had underwent occupational specialty training (28.0%). Except for the education levels of the middle school graduate and the master’s degree, significant differences were found among the six groups. A large majority, 70.9%, of the participants reported they had attended courses on elder abuse, with the social workers most likely to have attended, and the medical doctors the least likely to have attended.

**Table 1:** Study samples’ characteristics by occupational category (*N* = 311).

Variable		Occupational category						Test statistics
	All (*N* = 311)	HVN (*n* = 60)	MD (*n* = 44)	CM (*n* = 51)	CW (*n* = 47)	PHN (*n* = 46)	SW (*n* = 63)	
Sex^a^, *N* (%)								χ2 = 112.2***
Male	99 (31.9)	1 (1.7)	41 (93.2)	10 (20.0)	15 (31.9)	7 (15.2)	25 (39.7)	
Female	211 (68.1)	59 (98.3)	3 (6.8)	40 (80.0)	32 (68.1)	39 (84.3)	38 (60.3)	
Age^a^ (min = 22, max = 77), *M* (*SD*)	47.3 (11.0)	47.9 (7.0)	58.3 (9.0)	51.1 (8.3)	48.0 (10.1)	40.8 (10.5)	40.4 (10.7)	*F* = 25.4***
Years of work related to care of the elderly^b^^,^ ^c^ (min = 0, max = 45), *M* (*SD*)	13.2 (7.5)	15.2 (7.5)	17.1 (9.6)	16.3 (5.1)	13.7 (4.1)	8.5 (6.6)	9.1 (6.2)	χ2 = 65.9***
Total years of experience^a^^,^ ^d^ (min = 1, max = 55), *M* (*SD*)	18.7 (9.7)	22.7 (7.4)	30.4 (9.4)	17.9 (6.1)	16.0 (7.5)	14.3 (9.2)	12.6 (7.4)	χ2 = 107.6***
Education level, *N* (%)								
Middle school graduate	2 (0.6)	0 (0)	0 (0)	1 (2.0)	1 (2.1)	0 (0)	0 (0)	χ2 = 4.4
High school graduate	28 (9.0)	1 (1.7)	0 (0)	8 (15.7)	17 (36.2)	0 (0)	2 (3.2)	χ2 = 60.6***
Underwent occupational specialty training	87 (28.0)	47 (78.3)	0 (0)	11 (21.6)	12 (25.5)	11 (23.9)	6 (9.5)	χ2 = 104.8***
Junior college graduate	43 (13.8)	6 (10.0)	0 (0)	18 (35.3)	8 (17.0)	9 (19.6)	2 (3.2)	χ2 = 35.2***
Four-year/Six-year university graduate	134 (43.1)	6 (10.0)	30 (68.2)	12 (23.5)	9 (19.1)	24 (52.2)	53 (84.1)	χ2 = 101.8***
Master’s degree	4 (1.3)	0 (0)	1 (2.3)	1 (2.0)	0 (0)	2 (4.3)	0 (0)	χ2 = 6.1
Doctoral degree	13 (4.2)	0 (0)	13 (29.5)	0 (0)	0 (0)	0 (0)	0 (0)	χ2 = 82.3***
Attended courses on elder abuse^a^^,^ ^d^, *N* (%)								χ2 = 120.4***
No	90 (29.1)	29 (48.3)	38 (86.4)	3 (6.0)	13 (27.7)	3 (6.5)	4 (6.5)	
Yes	219 (70.9)	31 (51.7)	6 (13.6)	47 (94.0)	34 (72.3)	43 (93.5)	58 (93.5)	

HVN, home-visit nurse; MD, medical doctor; CM, care manager; CW, care worker; PHN, public health nurse; SW, social worker.

***p < .000, Calculated using chi-squared test for categorical variables, one-way ANOVA or Kruskal-Wallis test for continuous variables.

a. One CM not included.

b. Two MDs not included.

c. One PHN not included.

d. One SW not included.

### Differences in knowledge, attitudes, and behaviors among the six groups

Table 2 shows means (SD) and differences in knowledge, attitudes, and behaviors about elder abuse among the six groups. The Kruskal-Wallis analysis revealed significant differences in the knowledge, X^2^ (5, *N* = 311) = 60.8, *p* < .001, and attitudes, X^2^ (5, *N* = 311) = 69.9, *p* < .001, toward elder abuse among the six groups. Post hoc comparisons using Steel-Dwass tests indicated that medical doctors scored significantly lower than other groups, with the exception of home-visit nurses, regarding knowledge of elder abuse. Considering attitudes toward elder abuse, the Steel-Dwass tests showed that although no difference was found between public health nurses and social workers, both groups scored significantly higher than the other four groups, which present a more positive attitude toward knowledge of elder abuse. In addition, the one-way ANOVA indicated a statistically significant difference in the behaviors for dealing with elder abuse among the six groups, F (5,137) = 19.7, *p* < .001. Post hoc comparisons using Tukey’s tests showed lower mean behavior scores for home-visit nurses, medical doctors, and care workers compared to those for care managers, public health nurses, and social workers.

**Table 2:** Differences in knowledge, attitudes, and behaviors regarding domestic elder abuse among healthcare workers (*N* = 311)^a^.

Variable	Occupational category						Test statistics
	HVN	MD	CM	CW	PHN	SW	
Knowledge^b^ *M* (*SD*)	22.8 (6.2)	19.4 (6.1)	25.6 (5.1)	24.1 (6.0)	28.1 (3.6)	26.6 (5.1)	χ2 = 60.8 (*p* < .001)
	HVN < PHN (*p* < .001) and SW (*p* = .003)	MD < CM (*p* < .001), CW (*p* = .007), PHN (*p* < .001) and SW (*p* < .001)	CM > MD (*p* < .001)	CW > MD (*p* = .007), and CW < PHN (*p* = .010)	PHN > HVN (*p* < .001), MD (*p* < .001) and CW (*p* = .010)	SW > HVN (*p* = .003) and MD (*p* < .001)	
Attitudes^b^ *M* (*SD*)	95.0 (6.7)	93.2 (6.4)	97.0 (6.9)	92.5 (6.9)	101.4 (8.4)	102.4 (8.1)	χ2 = 69.9 (*p* < .001)
	HVN < PHN (*p* < .001) and SW (*p* < .001)	MD < PHN (*p* < .001) and SW (*p* < .001)	CM > CW (*p* = .021), CM < PHN (*p* = .024) and SW (*p* = .018)	CW < CM (*p* = .021), PHN (*p* < .001) and SW (*p* < .001)	PHN > HVN (*p* < .001), MD (*p* < .001), CM (*p* = .024) and CW (*p* < .001)	SW > HVN (*p* < .001), MD (*p* < .001), CM (*p* = .018) and CW (*p* < .001)	
Behaviors^c^ *M* (*SD*)	78.0 (29.7)	76.8 (33.9)	97.4 (25.2)	70.2 (33.9)	105.8 (20.5)	110.6 (21.9)	*F* = 19.7 (*p* < .001)
	HVN < CM (*p* = .004), PHN (*p* < .001) and SW (*p* < .001)	MD < CM (*p* = .005), PHN (*p* < .001) and SW (*p* < .001)	CM > HVN (*p* = .004), MD (*p* = .005) and CW (*p* < .001)	CW < CM (*p* < .001), PHN (*p* < .001) and SW (*p* < .001)	PHN > HVN (*p* < .001), MD (*p* < .001) and CW (*p* < .001)	SW > HVN (*p* < .001), MD (*p* < .001) and CW (*p* < .001)	

HVN, home-visit nurse; MD, medical doctor; CM, care manager; CW, care worker; PHN, public health nurse; SW, social worker.

a. Consisting of 60 HVNs, 44 MDs, 51 CMs, 47 CWs, 46 PHNs, and 63 SWs.

b. Differences in knowledge and attitudes were analyzed by Kruskal-Wallis and Steel-Dwass tests.

c. Difference in behaviors was analyzed by one-way ANOVA and Tukey’s tests.

### Variables predicting knowledge, attitudes, and behaviors regarding elder abuse

The results obtained from the stepwise multiple regression analyses are presented in Table 3. Multiple regression analysis showed that the demographic factor of age had a significant negative influence upon knowledge of elder abuse (β = -.13, *p* = .034), with higher age associated with lower knowledge of elder abuse. In terms of sex, females showed significantly more positive attitudes toward elder abuse than males (β = .11, *p* = .031). In addition, the length of work related to the care of elderly also significantly influenced the attitudes (β = .14, *p* = .011), with longer years of work related to the care of elderly associated with positive attitudes toward elder abuse. In terms of behaviors, the only demographic factor showing a significant influence (β = .20, *p* < .001) was the years of work related to the care of elderly, with a longer duration work caring for the elderly associated with actively dealing with elder abuse. In addition, medical doctors showed significantly lower influence with regard to knowledge about elder abuse than did home-visit nurses, care managers, care workers, public health nurses, and social workers. Regarding the attitudes toward elder abuse, care managers, public health nurses, and social workers were influenced to a significantly stronger degree than were medical doctors, and they also reported significantly stronger influence on behaviors in dealing with elder abuse than did medical doctors. The R^2^ values of the multiple regression model for knowledge, attitudes, and behaviors were .24, .26, and .28, respectively.

**Table 3:** Variables predicting healthcare workers’ knowledge, attitudes, and behaviors regarding domestic elder abuse (*N* = 302)^a^.

	Knowledge			Attitudes			Behaviors		
Independent variable	B	β	*p*	B	β	*p*	B	β	*p*
Age	-0.07	-.13	.034						
Sex (Reference: male)				1.96	.11	.031			
Years of work related to care of elderly				0.15	.14	.011	0.84	.20	< .001
Occupation (Reference: MD)									
HVN	2.92	.19	.010						
CM	6.02	.36	< .001	3.64	.16	.003	22.33	.26	< .001
CW	4.26	.26	< .001						
PHN	7.84	.47	< .001	8.56	.37	< .001	36.31	.41	< .001
SW	6.46	.43	< .001	9.96	.49	< .001	41.44	.56	< .001
R^2^	.24 (*p* < .001)			.26 (*p* < .001)			.28 (*p* < .001)		

HVN, home-visit nurse; MD, medical doctor; CM, care manager; CW, care worker; PHN, public health nurse; SW, social worker. Analyzed using stepwise multiple regression analyses.

a. Consisting of 60 HVNs, 42 MDs, 47 CMs, 47 CWs, 45 PHNs, and 61 SWs.

### Examination of the KAB Model

**Fig 1:**
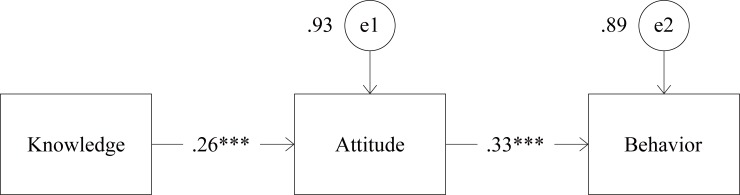
Examination of the knowledge-attitude-behavior (KAB) model (*N* = 311)^a^. Fit index: GFI = .99, AGFI = .96, RMSEA = .08, NFI = .95, CFI = .96. ***p < .001, e1 and e2 denote residual variance of the observed. a. Consisting of 60 home-visit nurses, 44 medical doctors, 51 care managers, 47 care workers, 46 public health nurses, and 63 social workers.

The result of the examination of the KAB model is presented in Fig 1. The final model showed a strong goodness-of-fit with GFI = .99, AGFI = .96, RMSEA = .08, NFI = .95, and CFI = .96. Knowledge (β = .26, *p* < .001) had direct positive effects on attitude, and attitude (β = .33, *p* < .001) also showed direct positive effects on behavior.

## Discussion

To our knowledge, no previous study has assessed healthcare workers’ knowledge, attitudes, and behaviors regarding elder abuse in Japan. This study therefore provides a preliminary insight into their perceptions. The results revealed that public health nurses and social workers had the most astute perceptions of elder abuse, believed mainly because social workers are positioned as professionals who have dealt with elder abuse since the establishment of the specialist team in dealing with domestic elder abuse through cooperative ties between the Japanese Association of Certified Social Workers (JACSW) and the Japan Federation of Bar Associations (JFBA) in 2006 [25]. The social workers’ extensive experience related to elder abuse enables them to recognize elder abuse as a common and important problem more readily than other health professionals such as nurses and medical doctors [26]. The public health nurse who works in the community is also expected to assess and manage elder abuse cases [15]. They have the responsibility to utilize and support various social resources for the elderly, their caregivers, and their family, as well as supporters such as care managers, in their consideration of threats to the well-being of elderly people. Over the past few years, although health and welfare professionals have established a network aimed at eliminating abuse of elderly, elder abuse is still mainly dealt with by public health nurses and social workers, especially those who work in the Comprehensive Community Care Centers. Because of this current situation in Japan and the roles and responsibilities borne by social workers and public health nurses, these individuals may have a more positive attitude toward knowledge of elder abuse than other healthcare workers.

Medical doctors were found to have the lowest level of knowledge about elder abuse and its acts. This reflects a phenomenon in Japan similar to that of Britain where, in spite of their greater contact time with patients, therefore supposedly placing them in a comparatively better position to detect abuse, medical doctors had difficulty in recognizing suspected cases of elder abuse [27]. Medical doctors had a significantly lower level of knowledge compared to care managers, care workers, public health nurses, and social workers. This may be partially because education for medical doctors currently focuses almost exclusively on medical care and physical assessment, while the topic of elder abuse is not covered. In the future therefore, education concerning elder abuse should be integrated into the curriculums for medical doctors at Japanese universities. Moreover, although attendance in courses on elder abuse was not found as a predictive variable of knowledge, as courses on elder abuse can somewhat help healthcare workers in understanding the salient points of the issue of abuse and knowing what action to take following discovery, it will be necessary to actively encourage more physicians to attend courses on elder abuse, as this group was least frequently represented among healthcare workers. Although home-visit nurses tended to have a better level of knowledge than medical doctors, no significant difference was found between them, in line with research by Almogue, et al. [28] which indicated that both nurses and physicians have inadequate levels of knowledge regarding elder abuse issues.

Previous research has found that nurses and medical doctors tend to not detect and report elder abuse out of beliefs that elder abuse is a private family matter [29]. Moreover, although care management overseen by care managers is indispensable for the prevention or reduction of abuse of elderly people, Omote and Saeki have pointed out that care managers feel reluctant to acknowledge abuse and report difficulties in implementing suitable care management for elderly people [30]. These difficulties, encountered by home-visit nurses, medical doctors, and care managers, might foster relatively negative attitudes toward knowledge of elder abuse. Despite its aging population, Japan currently faces a shortage of care workers and this results in excessive burdens on current care workers. Because overwork has been shown to be an important factor in burnout among care workers [31], and attitudes condoning abuse were correspondingly higher when burnout was a factor [32], we suggest that the relatively negative attitudes toward knowledge of elder abuse by the care workers in this study may to some degree be attributable to burnout due to overwork.

Besides public health nurses and social workers, care managers were found to espouse more positive behaviors with regards to aims to tackle elderly abuse compared to those by home-visit nurses, medical doctors, and care workers. This may be because care managers have a role in formulating the care plan for people receiving long-term care insurance and are constantly in contact with the elderly. When a case of elder abuse is reported, care managers always participate in a meeting with public health nurses and social workers to obtain a grasp of the current status of elder abuse and formulate an appropriate care plan that can alleviate as well as eliminate the situation of abuse. On the other hand, as home-visit nurses, medical doctors, and care workers have more emphasis on medical care and prevention of physical injury of the elderly, even though they might report cases of elder abuse, it is common for them to leave the most of the work of dealing with elder abuse to social workers, public health nurses, and care managers. Therefore, it may be necessary to consider countermeasures as well as to review the role of each healthcare worker within multi-disciplinary collaboration teams aiming to eliminate abuse of the elderly so as to make medical doctors, home-visit nurses, and care workers more actively involved in dealing with elder abuse.

Our results also demonstrated that the healthcare workers’ age negatively influenced their knowledge of elder abuse. This may be because in Japan, since the 1990s, elder abuse has come to attract public attention resulting in the initiation of education on elder abuse for nursing and welfare students. Moreover, since the number of complaints and incidents reported on elder abuse has continued to rise despite the enforcement of the Elder Abuse Prevention Model Project by the Ministry of Health, Labour and Welfare in 2005 and the “Elder Abuse Prevention Law” (Act on the Prevention of Elder Abuse, Support for Caregivers of Elderly Persons and Other Related Matters) in 2006, education on elder abuse has received increasingly higher priority, with numerous universities and nursing care insurance facilities including education on elder abuse in their basic curriculums. As such, the degree of knowledge about elder abuse is negatively influenced by the healthcare workers’ age. This also highlights the need for continuing education on elder abuse, to expand the knowledge about elder abuse of healthcare workers of a wide range of ages. Sex was a significant predictor of the healthcare worker’s attitudes toward elder abuse, with female healthcare workers showing more positive influence. This may be explained in several ways. Firstly, in Japan, among the abused elderly 76.8% were females [19]; this may make female healthcare workers more willing to devote themselves to dealing with elder abuse within the consideration of helping a person of the same sex. The study on gender differences in attitudes of healthcare staff toward violence against women also found that female primary healthcare workers tended to have a more critical attitude toward violence against women than did male staff [33]. Secondly, research on sex differences in emotionality has concluded that women in general tend to be more emotionally expressive than men [34, 35]. From this point of view, we could argue that when faced with concerns about abuse, female healthcare workers may be better equipped to recognize the emotions of elderly people that may be indicative of abuse, and more easily express their motivations to try to help the abused elderly individual. Positive emotional expression may be reflected in attitudes toward elder abuse, as was pointed out by Altmann that attitude structure can be described in terms of three components: affective, behavioral, and cognitive [18]. In addition, the finding that years of work related to the care of the elderly had a positive influence on healthcare workers’ attitudes and behaviors regarding elder abuse may be related to the fact that the longer the years of work, the greater the likelihood of encountering cases of elder abuse. As a result, a richer experience of cases of elder abuse leads to more positive attitudes and strategies for actively responding to elder abuse.

The results here indicate that the KAB model is useful for predicting healthcare workers’ perceptions of elder abuse. The healthcare workers’ knowledge had direct effects on their attitude and indirect influence on their behavior through attitude. This corresponds with previous studies showing that knowledge and attitudes can affect the way in which healthcare workers deal with elder abuse [36, 37]. Because the KAB model is effective in predicting healthcare workers’ perceptions of elder abuse, it is possible to use it as a framework for education on elder abuse for healthcare workers. To improve healthcare workers’ behavior in dealing with elder abuse, it is important to increase their knowledge of elder abuse to change their attitudes in a positive direction. It is also essential to directly address such attitudes because of their direct effects on behavior.

The present findings should be interpreted with caution owing to several limitations. Firstly, as participants were recruited in two city wards, irrespective of their number their scope might not fully reflect the entire population, which might influence the generalization of the findings. Secondly, the research tools were extracted from different existing literature regarding elder abuse and the response options of these questions were different. Such integration, therefore, might have a negative influence on reliability and validity. Thirdly, because significant differences have been found in healthcare workers’ knowledge, attitudes, and behaviors, it may be necessary to confirm the KAB model’s fitness with elder abuse using stratified sampling based on healthcare workers’ occupational category. However, because the sample size of each healthcare worker type in this study is too small to permit assessment, a study with a larger sample size may be required to verify the KAB model’s fitness.
