# Correction: Comparison of perceptions of domestic elder abuse among healthcare workers based on the Knowledge-Attitude-Behavior (KAB) model

**DOI:** 10.1371/journal.pone.0210916

**Published:** 2019-01-14

**Authors:** Qinqiuzi Yi, Naohiro Hohashi

There is an error in affiliation 1 for authors Qinquizi Yi and Naohiro Hohashi. Affiliation 1 should be: Division of Family Health Care Nursing, Graduate School of Health Sciences, Kobe University, Japan.

## References

[1] Yi Q, Hohashi N (2018) Comparison of perceptions of domestic elder abuse among healthcare workers based on the Knowledge-Attitude-Behavior (KAB) model. PLoS ONE 13(11): e0206640 10.1371/journal.pone.0206640 30383824 PMC6211737

